# **Incidence of retinopathy in chronic hepatitis C patients treated with pegylated interferon alpha 2a and ribavirin combination therapy**

**DOI:** 10.12669/pjms.311.6321

**Published:** 2015

**Authors:** Muhammad Kashif, Muhammad Khurram Saleem, Imran Khan Farooka, Amina Husnain, Arif Mahmood Siddiqui

**Affiliations:** 1 **Dr. Muhammad Kashif, Registrar Medicine,Medical Unit II, Jinnah Hospital Lahore, Pakistan.**; 2 Dr. Muhammad Khurram Saleem, Assistant Professor of Medicine, CMH Lahore Medical College, Combined Military Hospital Lahore, Pakistan.; 3 Dr. Imran Khan Farooka, Senior Registrar Medicine, Medical Unit II, Jinnah Hospital Lahore, Pakistan.; 4 Dr. Amina Husnain, Assistant Professor of Medicine, Medical Unit II, Jinnah Hospital Lahore, Pakistan.; 5 Prof. Dr. Arif Mahmood Siddiqui, Head of Department, Medical Unit II, Jinnah Hospital Lahore, Pakistan.

**Keywords:** Chronic Hepatitis C patients, Pegylated interferon alpha 2a and ribavirin combination therapy, Retinopathy

## Abstract

**Objectives:**

The objective of this study was to determine the incidence of retinopathy in chronic hepatitis C patients treated with Pegylated interferon alpha 2a and Ribavirin.

**Methods:**

This descriptive case series study was conducted in Medical Unit II of the Jinnah Hospital Lahore from September 2012 to February 2013. One hundred chronic hepatitis C patients visiting Medical Unit II outpatient department fulfilling inclusion criteria were selected for this study via non probability purposive sampling. Patients were started on pegylated interferon and ribavirin combination therapy. Subjects were subjected to dilated eye fundoscopic examination at the start of therapy and then after three months of the therapy.

**Results:**

One hundred patients were included in this study. Out of these 100 patients 5% developed retinopathy whereas fundus examination was normal in rest of the patients.

**Conclusion:**

Interferon therapy can lead to retinopathy. Periodic fundoscopic examinations help in early detection and prevent progression to permanent visual loss.

## INTRODUCTION

Chronic hepatitis C affects more than 170 million people in the world^1^ and around 10 million people in Pakistan^2^ and may eventually lead to cirrhosis or hepatocellular carcinoma. Published guidelines recommend pegylated interferon and ribavirin combination therapy as a first line treatment. Use of interferon has been associated with various adverse effects including influenza like syndrome, toxicities of central nervous, gastrointestinal, cardiovascular, endocrine, urinary and musculoskeletal system.^1^^,^^3^ However ocular toxicity was not reported before the use of interferon in chronic hepatitis.

Combination therapy also effects ocular system. One of the ocular complications associated with combination therapy in hepatitis C patients is retinopathy.^1^^,^^3^^-^^8^ Interferon associated retinopathy was first reported in 1990 in a 39 year old patient who developed retinal hemorrhages and cotton wool spots.^1^

Retinopathy is characterized by cotton wool spots, retinal hemorrhages and macular edema. Other atypical ocular complications are ischemic optic neuropathy neovascular glaucoma, peri phlebitis, ocular nerve paralysis, optic disc edema, sub conjunctival, pre retinal and vitreous haemorrhage, retinal vein occlusion and pan opthalmitis.^4^^, ^^6^^,^^9^^,^^10^

Interferon is an anti inflammatory, anti viral, anti tumor and immunomodulatory cytokine.^4^ Ribavirin is an antiviral agent having immunoregulatory activity.^4^ Exact mechanism responsible for retinopathy is not clear. Postulated mechanisms include immune complex deposition at vessel, immunological dysfunction and adherence to vessel wall^4^, disturbance in retinal microcirculation.^1^ Hepatitis C associated vasculitis may also play a role in the development of ischemic retinopathy.^3^

Diabetes mellitus, hypertension, patient age, dose of interferon are considered to be the possible risk factor for the development of retinopathy.^1^^,^^3^^,^^5^^,^^6^ Similarly use of combination therapy including interferon and ribavirin is associated with increased incidence of retinopathy than with interferon monotherapy.^1^

It is believed that retinopathy may develop as early as by 12 weeks after the start of treatment and disappear in about 4 weeks during the course of therapy.^1^ It is usually asymptomatic but can lead to serious complications such as loss of vision.

Incidence of retinopathy in hepatitis C patients treated with Interferon Ribavirin therapy is approximately 16%^3^^,^^4^^,^^6^ in different studies. No comprehensive data regarding the incidence of retinopathy in chronic hepatitis C patients receiving interferon and ribavirin therapy is available in Pakistan. As large number of Pakistani population is suffering from hepatitis C and taking interferon-ribavirin combination therapy so it is highly desirable to determine the incidence of retinopathy in these patients. Retinopathy can be detected by fundoscopic examination which is very easy and cheap to perform. Periodic fundoscopic examination of chronic hepatitis C patients undergoing combination therapy with interferon and ribavirin may help in early detection and prevention of this dreadful complication which can even result in permanent blindness.^4^^, ^^5^^, ^^8^

## METHODS

This descriptive case series study was conducted in Medical Unit II of Jinnah hospital Lahore. Sample size of 100 cases was calculated with 95% confidence level, 7.5% margin of error and taking expected percentage of Interferon induced retinopathy i.e. 16% in patients of chronic hepatitis C on pegylated interferon and ribavirin combination therapy for three months. Non probability purposive sampling was used and the study lasted for six months.

***Operational Definitions:***

***Chronic Hepatitis C patients:*** Patients with quantitative PCR detected for HCV RNA.

***Retinopathy:*** Presence of any one of the following mentioned below will be labeled as retinopathy^9^: Presence of cotton wool spots, retinal haemorrhage and macular edema on dilated eye examination with ophthalmoscope after three months of the start of interferon and ribavirin combination therapy.

***Pegylated Interferon and Ribavirin combination therapy: ***Pegylated interferon alpha 2a in the dose of 180ugm, s/c once weekly for 6 months and Ribavirin in the dose of 800 to 1200mg daily for 6 months.

***Inclusion Criteria:*** Both males and females. Age between 18 to 60 years. Chronic Hepatitis C patients as per operational definition enrolled for pegylated interferon and ribavirin combination therapy and Patients who don’t have retinopathy on baseline fundoscopic examination at the start of interferon and ribavirin combination therapy.

***Exclusion Criteria:*** Diabetic patients i.e. patients with history of diabetes taking insulin or oral hypoglycemic agents and patients having blood sugar fasting >126mg/dl on initial baseline laboratory test for unknown reason. Hypertensive patients i.e. with history of hypertension taking anti hypertensive medicines and patients having BP >160/90 mmHg on initial clinical examination on two or more occasion for unknown cases. Retreatment cases i.e. patients who are non responders to interferon therapy or relapsed cases who have already taken interferon and ribavirin combination therapy on basis of available medical record. Patients receiving interferon therapy for any other diseases like multiple sclerosis.

***Data Collection Procedure:*** After an informed consent, patient’s demographic data including name, age, and gender were noted. They were started on pegylated interferon alpha 2a and ribavirin combination therapy. A dilated eye fundoscopic examination was performed after 3 months of the start of combination therapy to assess retinopathy (as per operational definition). All the data was collected on separate patients Performa.

***Statistical Method:*** The SPSS software version 18.0 was used to analyze the data in the form of tables and graphs. Age is presented as mean and standard deviation. Gender and presence or absence of retinopathy is presented as frequency and percentage. Data is stratified for age to address effect modifiers.

## RESULTS

This study was conducted on 100 patients in which, 52 were male and 48 were females. Their mean age was 35 years, among males the mean age was 33.8846 z+9.16S.D (95% C.I =31.334-36.4348) where as in females the mean age was 36.3125+9.18 (95% C.I = 33.6464-38.9786). Out of 100 patients, 5% patients were found to have retinopathy whereas in 95% patients, fundus examination was normal.

**Table-I:** Frequency of Retinopathy positive and negative patients

**Retinopathy**	**Frequency**	**Percentage**
Negative	95	95.0
Positive	05	5.0
Total	100	100.0

**Table-II:** Frequency of Retinopathy according to gender

**Gender**	**Retinopathy negative**	**Retinopathy positive**	**Total**
Male	48	4	52
Female	47	1	48
Total	95	5	100

**Table-III:** Frequency of retinopathy according to age group

**Age**	**Retinopathy negative**	**Retinopathy positive**	**Total**
20-27	19	0	19
28-33	30	1	31
34-39	16	1	17
40-60	30	3	33
Total	95	5	100

## DISCUSSION

Combination therapy of pegylated alpha 2a interferon and ribavirin is currently the most successful treatment of patients with chronic hepatitis C. However the patients must be monitored closely, as this therapy can lead to serious ocular and systemic side effects.

Retinopathy is a well recognized side effect of this therapy and is characterized by cotton wool spots, retinal hemorrhages and cytoid macular edema. These changes occur most notably around the optic nerve head and in the posterior pole.^10^ Although most of them resolve and are asymptomatic, serious complications like macular edema can lead to permanent visual loss. Retinopathy usually appears by 12 weeks and usually resolves by end of treatment.^11^ Therefore baseline and ongoing assessment by ophthalmologists have been advocated in various studies.

In this study, 5 out of 100 i.e. 5% patients developed retinopathy when seen after three months of the start of combination interferon and ribavirin therapy. In previous studies reported frequency of retinopathy with interferon therapy ranged from 6 to 26%. In one study Ansari N et al.^12^ in 2010 reported 14.6% of chronic hepatitis C with no co morbid illness developed retinopathy on interferon ribavirin combination therapy. Ansari N et al.^12^ further reported that 58% of diabetic chronic hepatitis C patients and 60% of hypertensive chronic Hepatitis C patients developed retinopathy with interferon- ribavirin combination therapy. This type of study was also conducted by Cuthbertson FM et al.^6^ in 2004 and they found retinopathy in 15.5% of the patients. Similar type of study was also conducted by Okuse C et al.^1^ and Narkewicz MR et al.^13^ and both found retinopathy in 19% of the patients.

In this study 5% of the patients developed retinopathy which is considerably lower than the studies mentioned above. The main reason for this low frequency is that in our study those chronic hepatitis C patients were selected in whom there were no co morbid illness, and risk factors for retinopathy like diabetes mellitus and hypertension were excluded.

This and previous studies prove that retinopathy is a well known complication of interferon and Ribavirin therapy and chronic hepatitis C patients undergoing this therapy should be closely monitored and when cotton wool spots encroach macula or cytoid macular edema is present, one should consider termination of interferon therapy.^14^

## CONCLUSION

Interferon therapy can lead to retinopathy. Periodic fundoscopic examinations help in early detection and prevent progression to permanent visual loss.

## Authors Contribution:

All the authors mentioned above equally contributed in drafting this manuscript including conception and design of article, data collection and analysis, reviewing final draft, searching appropriate references and approved the final manuscript.
